# When the Room Is Spinning: Experience of Vestibular Neuritis by a Neurotologist

**DOI:** 10.3389/fneur.2020.00157

**Published:** 2020-03-03

**Authors:** Ji-Soo Kim

**Affiliations:** ^1^Department of Neurology, Seoul National University College of Medicine, Seoul, South Korea; ^2^Department of Neurology, Dizziness Center, Clinical Neuroscience Center, Seoul National University Bundang Hospital, Seongnam-si, South Korea

**Keywords:** vestibular neuritis, dizziness, vertigo, imbalance, nystagmus, head impulse tests

## Abstract

Vestibular neuritis (VN) is the most common cause of acute prolonged spontaneous vertigo, and is characterized by acute unilateral vestibular hypofunction, probably due to inflammation of the vestibular nerve. VN is diagnosed at the bedside when there is spontaneous horizontal-torsional nystagmus beating away from the side of the lesion, abnormal head impulse tests for the semicircular canals involved on the lesion side, and when other neurological symptoms and signs are absent. Here, as a neuro-otologist, I describe my experience during an attack of VN and discuss how it may help physicians to better understand why and what a patient feels during attacks of vertigo.

## Introduction

Vestibular neuritis (VN) is characterized by acute prolonged spontaneous vertigo due to unilateral peripheral vestibular hypofunction (1). Dix and Hallpike first coined the term “vestibular neuronitis” in 1952 to distinguish it from Meniere's disease even though the clinical features of VN had been described previously (2). VN as the most common cause of acute prolonged spontaneous vertigo, accounts for 3.2–9% of the patients visiting a dizziness clinic (3, 4), and has an incidence of ~3.5 per 100,000 population (5).

VN typically presents with acute dizziness/vertigo, nausea/vomiting, oscillopsia (illusory movement of the environment), and unsteadiness (6–8). VN is diagnosed confidently at the bedside when the patient has a spontaneous horizontal-torsional nystagmus beating away from the side of the lesion, abnormal head impulse tests (HITs) for the semicircular canals involved on the lesion side, and other neurological symptoms and signs are absent (9). Since HITs can be normal in patients with a weakness on caloric testing of <40%, the gold standard for detection of vestibular hypofunction of the horizontal (lateral) semicircular canal (HC) is still bithermal caloric irrigation (10, 11).

One of the long traditions in medicine is for physicians to write about their own infirmities (12–15). Following on this genre, I describe what a neuro-otologist experienced during an attack of VN and discuss its implications for both the patients and the treating doctors. Even though most of the symptoms and findings in VN have been well-recognized, I hope this personal experience helps one appreciate what is felt by the patients during the attacks of vertigo, as well as raises some questions about our understanding of VN.

## Case Description

I, a 54-year-old neuro-otologist, felt dizzy and nauseated on awakening in November, 2018. When I sat up and opened my eyes, I noticed that the room appeared to be continuously spinning to the left around the yaw axis (approximately top of the head to base of the skull), even when I was perfectly still. I had no headache, tinnitus, ear fullness, loss of hearing, or other symptoms. I had a history of hypertension for 3 years treated with medication. Other medical and family history was unremarkable.

Since one cannot observe one's own nystagmus in a mirror, I called my wife (a physician) and asked her to confirm whether I had nystagmus. The answer was yes. My hearing was the same in both ears and seemed normal with self-finger rubbing. As a neuro-otologist, I could instantly make a diagnosis of right VN. I was able to sit up but could not stand, so I crawled to the dressing room, and barely managed to change clothes with the help of my wife. When I tried to walk to go to the hospital, however, the vertigo and nausea immediately got worse, and I sank to the ground after a couple of steps due to severe vertigo, vomiting and associated epistaxis and could not walk even with help on both sides. I asked my wife to call 911 and was transferred to the nearby hospital by an ambulance and was admitted immediately.

During the preceding 2 weeks, I had a heavy social schedule every evening with moderate to heavy drinking. The day before I developed my prolonged vertigo, I had a vertigo spell while working in the afternoon outpatient clinic. The spell resolved within a minute. I had no precedent viral infection and otherwise felt well.

At admission, my vital signs and other findings on general physical examination were normal. I showed spontaneous nystagmus beating leftward even during visual fixation (Video 1). Bedside HITs were positive for the right HC, but I could not endure any further evaluation of vestibular function due to severe vertigo and nausea. During the first day in the hospital, I could not get up or eat anything due to severe nausea. I had to lie on my side, mostly to the left, since lying flat with my head facing up made the vertigo and nausea worse. However, there was no significant difference in how badly I felt depending on which ear was down when I was laying on my side. I was given intravenous normal saline 500 ml intermixed with metoclopramide 10 mg and diazepam 5 mg, which was continued to the next day. In the evening on the first day of my illness, I began to feel better, and a video recording showed a marked decrease in the intensity of my nystagmus.

**Figure 1:**
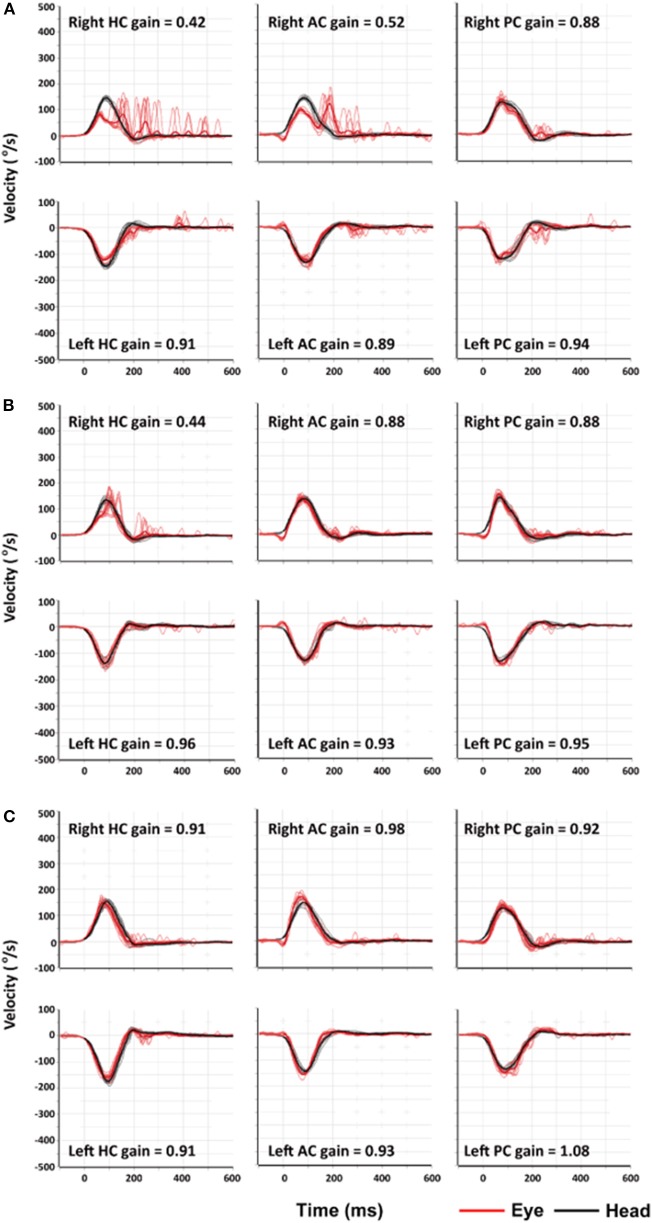
Findings of video-head impulse tests (video-HITs). **(A)** Four days after symptom onset, video-HITs showed decreased gains of the vestibulo-ocular reflex for the right horizontal (HC) and anterior semicircular canals (AC) while that for right posterior semicircular canal (PC) is normal. **(B)** Follow-up video-HITs, 10 days after symptom onset, showed a decreased gain only for right HC. **(C)** Two months after symptom onset, findings of video-HITs were normal.

The next day, I could sit and eat pieces of apple as the vertigo and nausea lessened, but I still could not walk. The spontaneous nystagmus and the sense of motion of the environment further decreased when looking straight ahead at a visual target. The nystagmus decreased during rightward gaze and increased during leftward gaze, obeying the Alexander's law, and, as is the case with peripheral vestibular lesions, increased when fixation was removed using Frenzel goggles (Video 2). The bedside HITs were still positive for the right HC with corrective catch-up saccades (Video 3). On the third day of my illness, I began to walk with assistance and was discharged. I began vestibular rehabilitation including rapid head movements to either side and walking. On the fourth day of my illness, I partially resumed my daily routine even though I still felt dizzy and unsteady. There was no longer a spontaneous nystagmus in straight ahead gaze with fixation of a target even though nystagmus could still be seen on leftward gaze and without visual fixation under the Frenzel glasses. Quantitative video-HIT showed decreased function of the vestibulo-ocular reflex (VOR) for right HC and anterior canal (AC) with the right posterior semicircular canal (PC) was spared (Figure 1A). The dizziness and unsteadiness lasted about a week more, especially provoked by moving. Thereafter the symptoms improved markedly, but I still noticed some illusory movements of the environment to the left when I rapidly turned my head to the right. I also noticed that occasionally I lurched to the right while walking. Ten days after the illness began, a follow-up video-HIT showed that only the HC had decreased function (Figure 1B). Some mild symptoms persisted for 1 more week, and then I felt normal, which was about 3 weeks after the illness began. Two months after the illness, the video-HIT was normal (Figure 1C).

## Discussion

### Symptoms and Signs

#### Vertigo

When my illness began in full force on awakening that morning, I felt nauseous and vertiginous with the room appearing to be spinning, which worsened over the day, and was especially uncomfortable whenever I moved. My symptoms were similar to those of most patients with VN who suffer from acute prolonged spontaneous vertigo with nausea and vomiting (1). The symptoms may develop suddenly or evolve over several hours, and occasionally there may be a brief prodrome within the few days before the onset of the full-blown syndrome (1, 16, 17). Vertigo is usually rotational, and is markedly aggravated by motion of the head. Most patients are quite uncomfortable with severe nausea and vomiting. When I opened my eyes the room seemed to be spinning to the left. Spontaneous nystagmus may give rise to illusion of apparent rotation of the environment (oscillopsia). Since this illusory rotation is generated by the slow phases of nystagmus and is in the opposite direction of the slow phases, spinning of the room to the left indicated left beating spontaneous nystagmus even though I could not see the nystagmus. Indeed, one cannot see one's own nystagmus in a mirror since the image of the eyes on the mirror move with the eyes. Thus, one must ask someone else to confirm the presence of nystagmus, or record the nystagmus using a video-cam and review the video clips later. Otherwise, one can also suspect that one has nystagmus by observing the drifting and resetting back to the straight ahead position of a target light from a charger or clock in darkness. One can also put one's fingers on the closed eyelid and feel the ocular bulb beating.

#### Prodromal Dizziness

Of interest, prodromal dizziness lasting a few minutes, in the few days just before the full onset of symptoms, may precede the prolonged spontaneous vertigo in as many as one fourth of patients with VN, just as I reported here (16, 17). These preceding episodes are mostly non-vertiginous dizzy attacks, often accompanied by nausea or unsteadiness. Prodromal attacks may develop abruptly or gradually (17). Recognizing this prodromal dizziness is important in VN when trying to differentiate VN from vertebrobasilar transient ischemic attacks. Thus, patients with a first transient spell of dizziness or vertigo should be warned that they may develop prolonged vertigo in the next few days. Indeed, the author saw a patient who had daily attacks of vertigo lasting several minutes for three consecutive days before he developed typical prolonged vertigo due to VN. The mechanism of this prodromal dizziness/vertigo in VN is unknown but may be due to an initial smoldering inflammation of the vestibular nerve. On the other hand, the hyperacute onset of dizziness can be the harbinger of an impending stroke.

#### Positional Preference

During the acute phase, I laid on my side, usually the left, because lying supine with my head pointed up made my vertigo worse. Even though I did not notice any difference in the severity of vertigo between the lying on either side, patients with VN usually prefer to lie in bed on the side with the healthy ear down and with their eyes closed (18). The mechanism of this positional preference is unknown though it implies an interaction between inputs from the otoliths and the semicircular canals (19, 20). Future studies should correlate the severity of the vertigo, the intensity of the nystagmus and the attitude of the head, as the function of the otoliths can now be relatively easily evaluated by looking for the ocular tilt reaction (OTR), tilt of the subjective visual vertical (SVV), and vestibular-evoked myogenic potentials (VEMPs) (21–23).

#### Balance

I could sit and stand with the feet apart during the acute phase. Even though patients with VN feel unsteady, they usually can sit or stand unaided with the feet apart since the information via the visual and somatosensory systems can still be used for balance control (18, 24, 25). Patients with VN tend to fall toward the lesion side while standing with the feet together or when trying to walk (26). Why I had such severe imbalance will be discussed below.

### Etiology

Although patients with VN may report a preceding or concurrent viral illness (2, 16), evidences of systemic viral infection as a cause of VN is unconvincing (16, 27). Indeed, I had no recent viral illness and no symptoms and signs of systemic infection. Instead, increasing evidence supports reactivation of latent type 1 herpes simplex virus (HSV-1) as a mechanism of VN, as has been proposed for idiopathic facial palsy (28). The possibility of a latent infection is supported by the detection of HSV-1 DNA in about two third of human vestibular ganglia on autopsy (28, 29). In mice, vestibular impairments with infected vestibular ganglion cells may be induced by inoculating HSV (30). Other mechanisms might include autoimmunity and microvascular ischemia of the vestibular labyrinth (4).

### Diagnosis

Since there are no confirmatory diagnostic tests, VN is a diagnosis of exclusion based on both the bedside and laboratory evaluation (Table 1) (8). While which structures in the labyrinth are affected can vary from patient to patient, the key symptoms and signs for VN include (1) acute prolonged vestibular symptoms, such as vertigo, nausea/vomiting and unsteadiness, (2) unidirectional horizontal-torsional nystagmus beating away from the lesion side, and (3) impaired semicircular function documented with HITs or caloric tests. The introduction of HITs and VEMPs makes it possible to evaluate all three semicircular canals, the utricle and the saccule, and has enabled defining the subtypes of VN (Table 2) (31–35).

**Table 1:** Clinical features of complete vestibular neuritis.

•Subacute or acute onset of spontaneous vertigo with nausea/vomiting. •Oscillopsia: illusory sensation of spinning of surroundings in the direction of nystagmus quick phase. •Horizontal-torsional spontaneous nystagmus beating toward the unaffected side. The nystagmus follows the Alexander's law, and is partially or totally suppressed under visual fixation. •Impaired function of the semicircular canals as revealed by head impulse tests or caloric testing. •Ipsiversive ocular tilt reaction (head tilt, skew deviation, and ocular torsion) and ipsiversive tilt of the subjective visual vertical/horizontal. The full picture of the ocular tilt reaction, however, is not very often seen clinically. •Decreased or absent responses of vestibular-evoked myogenic potentials (VEMP) during stimulation of the affected ear. VEMP responses might be normal or abnormal depending on the subtype. •Falling tendency toward the lesion side on standing or Romberg test/Ipsiversive rotation during Fukuda stepping test.

**Table 2:** Comparison of the findings among the subtypes of vestibular neuritis.

	**Superior**	**Inferior**	**Total**
SN	H(C)-T(C)-U	T(C)-D	H(C)-T(C)
HIT-AC	Impaired	Normal	Impaired
HIT-HC	Impaired	Normal	Impaired
HIT-PC	Normal	Impaired	Impaired
Caloric test	Abnormal	Normal	Abnormal
OTR	Ipsiversive	Normal	Ipsiversive
SVV	Ipsiversive	Normal	Ipsiversive
oVEMP	Abnormal	Normal	Abnormal
cVEMP	Normal	Abnormal	Abnormal

*AC, anterior semicircular canal; C, contraversive (quick phase); cVEMP, cervical vestibular-evoked myogenic potential; D, downbeat; H, horizontal; HC, horizontal semicircular canal; HIT, head impulse test; OTR, ocular tilt reaction; oVEMP, ocular vestibular-evoked myogenic potential; PC, posterior semicircular canal; SN, spontaneous nystagmus; T, torsional; U, upbeat; SVV, subjective visual vertical*.

**Figure 2:**
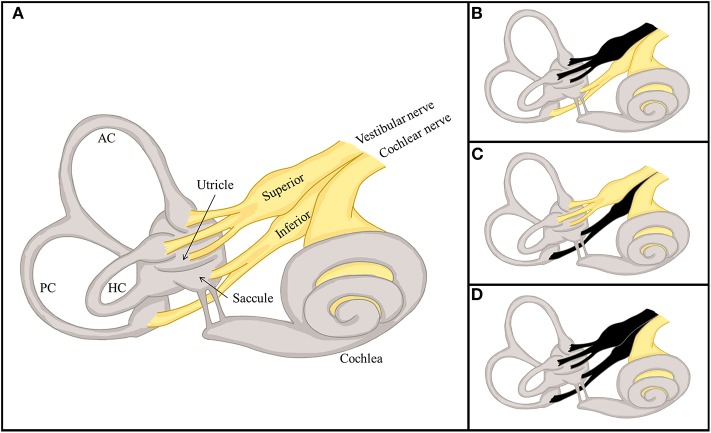
Divisional configuration of the labyrinth and 3 distinctive types of vestibular neuritis (VN). **(A)** The vestibular labyrinth may be subdivided into the superior and inferior divisions. The superior vestibular labyrinth comprises the anterior (AC) and horizontal semicircular canals (HC), and the utricle, and their afferents. In contrast, the inferior vestibular labyrinth consists of the posterior semicircular canal (PC) and saccule, and their afferents. **(B–D)** According to the divisions involved, VN may be classified into distinctive types, the superior **(B)**, inferior **(C)**, and total **(D)**.

The labyrinth can be divided into the superior and inferior divisions (Figure 2). The superior portion includes the AC, HC, utricle, and their afferents. In contrast, the inferior portion consists of the PC and saccule, and their afferents. According to which vestibular subdivisions are involved, VN may be classified into the superior, inferior, and total (superior + inferior) types. Superior VN is most common (42–100%), followed by total (15–56%), and inferior VN (2.3–15%) (35–42).

#### Spontaneous Nystagmus

The patterns of spontaneous nystagmus in VN depend on how much each semicircular canal is involved (20). When all three canals are equally affected, spontaneous nystagmus is horizontal-torsional, beating away from the lesion side (20). Spontaneous nystagmus in VN may also be accompanied by a vertical component that is usually upbeat reflecting the loss of AC function on one side since the AC is affected more than the PC (36). The horizontal nystagmus typically increases during the gaze in the direction of the quick phases, and decreases when looking in the opposite direction (Alexander's law) (18). Although spontaneous nystagmus can be seen with visual fixation especially during the acute phase, one must remove visual fixation with Frenzel glasses or infrared video goggles to see the true intensity and direction of the nystagmus (43). Indeed, no change or a decrease of spontaneous nystagmus with removal of visual fixation points to a central disorder. Thus, nystagmus should be evaluated with and without visual fixation. The spontaneous nystagmus typically increases after horizontal head shaking, during vibratory stimuli on the sternocleidomastoid muscle or skull, or with hyperventilation (44–46). These maneuvers are especially important during the subacute or chronic phase when spontaneous nystagmus becomes less conspicuous due to central compensation or some spontaneous recovery of labyrinthine function (46). Recording of and quantifying eye movements using oculography help defining the wave form of the nystagmus, and distinguishing it from that of central disorders or long-standing congenital nystagmus. The jerk-linear wave form is typical of peripheral vestibular disorders while other waveforms, such as jerk-velocity-decreasing, jerk-velocity-increasing and pendular, are either central or infantile (1, 47).

#### Head Impulse Tests (HITs)

The function of each semicircular canal can be evaluated at the bedside with the HIT (31). Bedside HIT has an acceptable sensitivity (11), but may appear negative when the vestibular deficits are mild (10, 11), or the corrective saccades, by which we infer hypofunction in the labyrinth, only occur during head impulses and cannot be seen on simple visual inspection (covert saccades) (48). Furthermore, evaluation of the vertical canals are often difficult to perform and the results are difficult to interpret (39). In these instances, quantitative HIT using video-oculography is necessary to infer the function of the semicircular canals (41, 49). The results of HITs may correlate with recovery of symptoms in VN (50). HITs were positive in 80% of patients with persistent dizziness in contrast to only 10% in those without dizziness.

#### Caloric Tests

Unilateral caloric paresis has been the diagnostic hallmark of VN (2). However, the caloric test can only evaluate the function of the HC, only in the lower frequency range of stimulation (~0.003 Hz) (1), and would also be normal when VN spares the HC, as in inferior VN (39).

#### Other Ancillary Tests

The OTR is comprised of head tilt, skew deviation, and ocular torsion (4). In VN, however, only a small percentage of patients have a complete OTR, and the skew deviation is usually smaller and transient compared to central lesions (50). A cross-cover test is used to detect a skew deviation, but when the skew is small and there is a spontaneous nystagmus, it may be hard for the examiner to see the skew deviation (25). Ocular torsion can also be quantified with fundus photography (4). In VN, the tilt of the SVV is the sensory manifestation of ocular torsion (51–53), and can be measured at the bedside using a simple, self-made bucket (54).

Even though results of VEMPs were unavailable in this report, cervical and ocular VEMPs can evaluate the function of the otoliths and help determine which division of the vestibular nerve is involved in VN (38, 55, 56). Patients with superior VN show abnormal ocular VEMPs but normal cervical VEMPs in response to air-conducted sound (ACS) (40). In contrast, patients with inferior VN show normal ocular VEMPs but abnormal cervical VEMPs in response to ACS (39, 40).

Inflammation of the vestibular nerve may be visualized on gadolinium-enhanced MRIs (57), but neuroimaging is mostly indicated when a central cause is suspected.

### Differential Diagnosis

In clinical practice, one must differentiate VN from other more serious disorders such as strokes (58–61). Acute unilateral peripheral vestibulopathy mimicking VN may also be caused by vascular insults to the vestibular labyrinth (60, 62). Isolated labyrinthine infarction remains a challenge since it cannot be confidently diagnosed even with current imaging techniques (61). However, isolated labyrinthine infarction is extremely rare, and usually the cochlea is also damaged (9, 63). Isolated infarction of the labyrinth may herald an infarction in the full territory of the anterior inferior cerebellar artery (AICA) (64). Infarctions involving the vestibular nuclei (65) or inferior cerebellum (59, 66, 67), may also mimic VN. Serial evaluation is often required since small infarctions involving the brainstem or cerebellum may go undetected with diffusion-weighted MRIs, especially during the acute phase (61, 64, 68). Multiple sclerosis may also involve the root entry zone of the eight nerve and mimic VN (69). Thus, the first question to be answered in patients with acute spontaneous vertigo and nystagmus is whether they have a peripheral VN or a central “vestibular pseudoneuritis” (61). It is not always possible to differentiate isolated vascular vertigo from acute peripheral vestibulopathy at the bedside. However, findings of neurotological examination including normal HITs, direction-changing nystagmus, and skew deviation (HINTS), and HINTS Plus (HINTS + hearing) point to a diagnosis of central vertigo with a high sensitivity and specificity (70, 71). These bedside tests are even more sensitive than early MRIs for diagnosing strokes (70, 72–75). Indeed, diffusion-weighted MRI is falsely negative in 12–20% of patients with an acute infarction (70, 75). Since mild skew deviation may not be detected when there is a spontaneous nystagmus, and gaze-evoked nystagmus may not develop in cerebellar strokes, HITs (and especially a negative HIT in the acute vestibular syndrome) are most useful for the differential diagnosis between isolated vascular vertigo and acute VN (70). Positive HITs, however, do not necessarily exclude central lesions (65, 67, 73, 76, 77). If HIT is normal, there is no need to perform tests of skew and nystagmus since one “red flag” is sufficient to suspect stroke. The other tests of the HINTS battery, skew and gaze-evoked nystagmus, are important for cases with positive HITs. Since the proportion of patients with skew devistion or gaze-evoked nystagmus is higher in AICA strokes, the sensitivity and specificity of HINTS are increased by applying all three tests (25). Because recurrence is rare in VN, an alternative diagnosis should be considered whenever patients have a second attack (78).

### Treatments

The treatments of VN include supportive care during the acute phase, medications targeting inflammation and viruses and vestibular rehabilitation, which will be discussed below.

#### Supportive Care

Symptomatic care with vestibular suppressants is used during the acute phase when the patients have severe nausea/vomiting and vertigo, but these medications should be avoided in the long-term since they may hinder central compensation (18). In patients with severe vomiting, intravenous fluids and anti-emetics may be needed to correct electrolyte imbalance and to avoid dehydration (18).

#### Steroids and Antiviral Agents

Given the shared theory of viral reactivation between VN and Bell's palsy, antiviral agents and steroids have been used (79, 80). However, there is currently insufficient evidence for giving corticosteroids to patients with VN (81). Valacyclovir alone or in combination with glucocorticoids was not effective, either (79).

#### Vestibular Rehabilitation

Vestibular exercises hasten vestibulospinal compensation significantly in patients with acute VN (82, 83). Balance training significantly reduces the time required for vestibulospinal compensation (84). Voluntary eye movements, active head movements, goal-directed movements, and walking should be encouraged to restore postural control and balance as soon as possible (83). Patients should exercise at least for 30 min several times a day.

### Course and Prognosis

I noticed nausea and vertigo on awakening, which worsened over a day, improved markedly over the following several days, and then resolved completely within 3 weeks of the beginning of the illness. Most patients with VN have subacute or acute spontaneous vertigo gradually increasing over several hours and reaching a peak within the first day (1). In most, the severe vertigo improves markedly within a day or two with residual symptoms gradually resolving over the following weeks (1, 46). Most of the recovery is due to central compensation rather than functional recovery in the affected ear (85). One sign of central compensation is reduction of spontaneous nystagmus, which requires about 3 weeks for resolution even though the dynamic responses of the VOR, as shown by the positive HIT, remained impaired (82). Central compensation to rid one of spontaneous nystagmus depends on restoration of balance between the level of spontaneous activity in the vestibular nuclei on either side (9, 86). While the symptoms from static vestibular imbalance invariably resolve over time, the symptoms from dynamic vestibular dysfunction tends to last longer or persist (46). In a previous study, the symptoms and signs of static vestibular imbalances (spontaneous nystagmus, ocular torsion, and ipsilesional SVV tilt) had mostly resolved by 3 months after the onset of the illness while the signs of dynamic vestibular imbalances (HIT, head-shaking nystagmus, vibration-induced nystagmus, and caloric paresis) were still present in more than 30% of the patients 1 year later (46). Given the earlier resolution of static vestibular imbalance (46), evaluation of dynamic vestibular dysfunction provides useful information about the degree of underlying vestibular imbalance in VN, especially during the chronic phase. In patients with an incomplete recovery of labyrinthine function, oscillopsia may be experienced during rapid head motion (83). The persistent imbalance that some patients have after acute VN may be due to many factors including inadequate central compensation, incomplete peripheral recovery, and psychophysiological and psychological features (87).

VN is known to recur only in 2–11% (78, 88). In 10–15% of patients with VN, BPPV may develop in the affected ear within a few weeks (89), which suggests a loosening of the otoconia due to labyrinthine inflammation. The second most important complication is persistent postural perceptual dizziness, which refers to persistent dizziness and unsteadiness associated with fear of falling, but without any real falls or vestibular dysfunction that can explain the symptoms (87, 90).

## Conclusion

Careful history taking and a focused neurotological examination are usually enough for diagnosis of VN. Imaging should be considered when there are findings inconsistent with VN since VN is a diagnosis of exclusion. The management of VN during the acute phase is mostly supportive, but vestibular rehabilitation should be initiated to hasten vestibular compensation even during the acute phase.

We can finally ask what insights or questions were raised by my experience with VN. Striking in my case was such severe imbalance to the point that I could not walk at all, even with considerable assistance. Normally this would be worrisome for a central process, which did not turn out to be the case for me. One might speculate that I also had severe involvement of both otolith organs though I did not seem to have an OTR. Also, puzzling was my “subconscious” preference to lie on one side but with no perceptual differences between the two in the degree of my vertigo. This finding points to both the difficulties of quantifying subjective sensations and the variability among human patients in their degree of subjective discomfort relative to objective findings on examination. All these observations point to a need for more testing of otolith function, including perhaps dynamic otolith function using head translations, combined with testing of all three semicircular canals to better understand the pathophysiology of vestibular diseases.

## Data Availability Statement

All datasets generated for this study are included in the article/Supplementary Material.

## Ethics Statement

Written informed consent was obtained from the patient for the publication of this case report.

## Author Contributions

J-SK designed and conceptualized the study, interpreted the data, and wrote the manuscript.

### Conflict of Interest

J-SK serves as an associate editor of Frontiers in Neuro-otology and on the editorial boards of the Journal of Clinical Neurology, Frontiers in Neuro-ophthalmology, Journal of Neuro-ophthalmology, Journal of Vestibular Research, Journal of Neurology, and Medicine.

## Supplementary Material

Video 1 The video clip in the morning on the first day of attack shows strong spontaneous left beating nystagmus even during visual fixation.

Video 2 On the second day of symptom onset, follow-up video recording shows a marked decrease in the intensity of spontaneous nystagmus in the primary gaze. The nystagmus increases during gaze in the direction of spontaneous nystagmus and decreases during gaze in the opposite direction (Alexander's law). The spontaneous nystagmus also increases with removal of visual fixation using Frenzel glasses, a typical pattern of vestibular nystagmus due to a peripheral lesion.

Video 3 Bedside head impulse tests were positive to the right with corrective catch-up saccades. The corrective catch-up saccades are small probably due to a small head excursion.
